# Structural/functional studies of Trio provide insights into its configuration and show that conserved linker elements enhance its activity for Rac1

**DOI:** 10.1016/j.jbc.2022.102209

**Published:** 2022-06-30

**Authors:** Sumit J. Bandekar, Chun-Liang Chen, Sandeep K. Ravala, Jennifer N. Cash, Larisa V. Avramova, Mariya V. Zhalnina, J. Silvio Gutkind, Sheng Li, John J.G. Tesmer

**Affiliations:** 1 Department of Medicinal Chemistry, University of Michigan, Ann Arbor, Michigan, USA; 2 Life Sciences Institute, University of Michigan, Ann Arbor, Michigan, USA; 3 Departments of Biological Sciences and of Medicinal Chemistry and Molecular Pharmacology, Purdue University, West Lafayette, Indiana, USA; 4 Department of Molecular and Cellular Biology, University of California-Davis, Davis, California, USA; 5 Department of Pharmacology and Moores Cancer Center, University of California, San Diego, San Diego, California, USA; 6 Department of Medicine, University of California San Diego, La Jolla, California, USA

**Keywords:** Trio, guanine nucleotide exchange factor (GEF), Dbl homology, pleckstrin homology, GTPase, structural biology, cryo-electron microscopy, hydrogen-deuterium exchange mass spectrometry, Rac, Rho, CT, Cral/Trio, DH, Dbl homology, EM, electron microscopy, FRET, Förster resonance energy transfer, GEF, guanine nucleotide exchange factor, HDX-MS, hydrogen-deuterium exchange mass spectrometry, MBP, maltose binding protein, PH, pleckstrin homology, RhoGEFs, Rho family guanine nucleotide exchange factors, SH3N, N-terminal Src homology 3 domain, SH3C, C-terminal Src homology 3 domain, TrioN, N-terminal GEF module, TrioC, C-terminal GEF module

## Abstract

Trio is a large and highly conserved metazoan signaling scaffold that contains two Dbl family guanine nucleotide exchange factor (GEF) modules, TrioN and TrioC, selective for Rac and RhoA GTPases, respectively. The GEF activities of TrioN and TrioC are implicated in several cancers, especially uveal melanoma. However, little is known about how these modules operate in the context of larger fragments of Trio. Here we show *via* negative stain electron microscopy that the N-terminal region of Trio is extended and could thus serve as a rigid spacer between the N-terminal putative lipid-binding domain and TrioN, whereas the C-terminal half of Trio seems globular. We found that regions C-terminal to TrioN enhance its Rac1 GEF activity and thus could play a regulatory role. We went on to characterize a minimal, well-behaved Trio fragment with enhanced activity, Trio_1284__–__1959_, in complex with Rac1 using cryo-electron microscopy and hydrogen-deuterium exchange mass spectrometry and found that the region conferring enhanced activity is disordered. Deletion of two different strongly conserved motifs in this region eliminated this enhancement, suggesting that they form transient intramolecular interactions that promote GEF activity. Because Dbl family RhoGEF modules have been challenging to directly target with small molecules, characterization of accessory Trio domains such as these may provide alternate routes for the development of therapeutics that inhibit Trio activity in human cancer.

Rho family guanine nucleotide exchange factors (RhoGEFs) (1) activate small guanosine triphosphatases (GTPases) of the Rho family (2), thereby regulating cell growth *via* transcriptional events and motility *via* modulation of the actin cytoskeleton. RhoGEF dysregulation is well known to lead to oncogenic phenotypes including growth, migration, and metastasis (3, 4). Trio (ARHGEF23) is an unusual member of the Dbl family of RhoGEFs in that it contains two catalytic RhoGEF modules (Fig. 1*A*) (5). Both RhoGEF activities of Trio are crucial to the growth and metastatic spread of uveal melanoma (6, 7), a particularly fatal cancer once it metastasizes to the liver and one with no effective therapeutics available (8, 9). In tumor xenograft models of metastatic uveal melanoma, knockdown of Trio is effective at reducing tumor size and weight, suggesting inhibitor molecules targeting Trio activity could serve as effective therapeutics (7). Trio can also be involved in adult T-cell leukemia (10) and is overexpressed in a variety of other cancers (7), leading to broad interest in targeting this protein.

**Figure 1:**
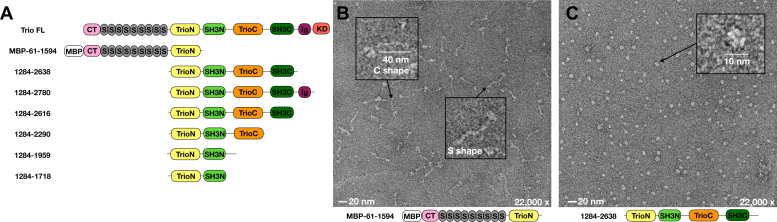
**Negative stain EM reveals low-resolution features of different regions of Trio.***A*, domain architecture of human Trio and variants used in this work. Domains are abbreviated as CT, Cral/Trio; S, spectrin repeat; TrioN, N-terminal GEF module; SH3N, N-terminal Src homology 3 domain; TrioC, C-terminal GEF module; SH3C, C-terminal Src homology 3 domain; Ig, immunoglobulin-like domain; KD, kinase domain; MBP, maltose binding protein. *B*, representative micrographs show that the N-terminal domains of Trio adopt an extended configuration, whereas the central region of Trio is condensed (*C*). Insets: representative particles with length estimates. EM, electron microscopy; GEF, guanine nucleotide exchange factor.

Dbl family GEF modules are composed of a Dbl homology (DH) domain that binds Rho GTPases followed by a regulatory pleckstrin homology (PH) domain. The N-terminal GEF module of Trio (TrioN) catalyzes nucleotide exchange on Rac subfamily members, whereas the C-terminal module (TrioC) catalyzes nucleotide exchange on RhoA subfamily members (5). Both TrioN and TrioC have been characterized structurally and functionally as standalone modules (11, 12, 13), and prior screening efforts have tried to identify molecules that target TrioC (14, 15, 16), but none have succeeded past *in vitro* studies. Understanding how the accessory regions and/or domains of Trio contribute to GEF activity may provide alternative therapeutic routes by which to modulate Trio function.

Most RhoGEFs are large, multidomain proteins, and in other RhoGEFs, accessory regions have been shown to positively or negatively regulate GEF activity. For example, p115-RhoGEF, PDZ-RhoGEF, and leukemia-associated RhoGEF are demarked by a regulator of G protein signaling homology domain that interacts with the heterotrimeric G protein Gα_12/13_ to positively regulate GEF activity in cells (17), whereas Asef, collybistin, and ephexin all contain adjacent SH3 domains that negatively regulate GEF activity (18, 19, 20). The functions of the accessory domains in Trio are primarily inferred by the roles of homologous domains in other proteins. The N-terminal Cral/Trio (CT) domain (Fig. 1) is homologous to Sec14 lipid transferase domains, which bind phosphoinositides or hydrophobic ligands (21, 22). One report further suggested that Dbl family CT domains could serve as a binding site for the G protein βγ (G_βγ_) heterodimer (23). The CT domain is followed by nine spectrin repeats (S1-S9), which are extended 3-helix bundles found in many proteins associated with the actin cytoskeleton and expected to serve a structural role (24). The TrioN GEF module is followed by a N-terminal Src homology 3 domain (SH3N), which canonically binds to proline-rich sequences containing the motif PXXP (25), as it does in Kalirin, a close homolog of Trio (26). Next is the TrioC module followed by another SH3 domain (C-terminal Src homology 3 [SH3C]). The next domain is an Ig-like domain that one report concluded binds to activated RhoA and thereby contributes to the localization of Trio on the plasma membrane (27). Trio ends with a protein kinase domain that belongs to the Ca^2+^·calmodulin kinase subfamily followed by an autoinhibitory C-terminal helical signature that interacts with Ca^2+^·calmodulin in functional members of the family. However, activity for the Trio kinase domain has yet to be reported.

Aside from the isolated functions of the individual Trio domains, it is also important to study how they function in context of the full-length protein. A region encompassing the spectrin repeats, TrioN GEF module, and SH3N domain has been shown as the minimal region of Trio necessary to stimulate neurite outgrowth in cells (28). In addition, the heterotrimeric G protein Gα_q_ has been shown to directly bind to the TrioC GEF module and stimulate RhoA exchange by relieving intramolecular autoinhibition within the TrioC module (13, 29, 30, 31). However, in cells where full-length Trio is present, Gα_q_ stimulates both TrioN and TrioC GEF activity (6, 7). It is unknown whether Gα_q_ signaling leads to activation of the TrioN module by mechanisms other than membrane recruitment.

In this study, we hypothesized that adjacent regions or domains could modulate the activity of the Trio GEF modules in either positive or negative ways, as implied by studies with the Kalirin SH3N domain (26) and be part of a larger regulatory framework controlled by Gα_q_. To test this theory, we purified various fragments of human Trio and compared their GEF activities, structures, and dynamics. Our negative stain electron microscopy (EM) data are consistent with the N-terminal half of Trio existing in an extended conformation dominated by the spectrin repeats and the middle of the protein containing both RhoGEF modules and their SH3 domains in a globular configuration, suggesting that these signaling domains and their extended linker regions could be closely associated. Our results are consistent with Gα_q_ regulating TrioC as a standalone module that is not obviously influenced by surrounding regions, but with TrioN being profoundly activated by conserved elements within a disordered region C terminal to the SH3N domain. However, these elements were not ordered in high resolution cryo-EM maps or evidenced in hydrogen-deuterium exchange mass spectrometry (HDX-MS) measurements. Our work thus further illuminates a network of intramolecular and intermolecular protein–protein interactions that underlie regulation in full-length Trio.

## Results

### Negative stain EM characterization of Trio fragments provides insights into overall architecture of Trio

To capture snapshots of the overall configuration of Trio, we first attempted to purify Trio fragments starting from the N terminus. The largest successfully purified to homogeneity (Trio_MBP-61-1594_) contained the CT domain, nine spectrin repeats, and TrioN. The protein was studied as the maltose binding protein (MBP) fusion because it aggregated when MBP was cleaved. Negative stain EM micrographs revealed an extended particle with ∼45 nm maximum length (Fig. 1, *B* and *C*). The Trio_MBP-61-1594_ particle (Inset, Fig. 1*B*) has a globular head, likely representing MBP and the CT domain, followed by a thin extended tail region of about 40 nm that likely corresponds to the spectrin repeat region of Trio. A fully extended length of 45 nm is estimated for the repeats from a model containing fully extended nine copies of the spectrin repeat. In comparison, the solution structure of the seven spectrin repeats of plectin is a rod-like structure of 35 nm, which would also correspond to 45 nm if there were nine repeats (32). In Trio, the shorter length may be accounted for by curvature and flexibility in the spectrin repeats. Some Trio_MBP-61-1594_ particles were for example shaped roughly like an “S” or a “C” (Fig. 1*B*, insets). The particle heterogeneity indicated that a 3D reconstruction using this N-terminal fragment would be challenging. Because longer Trio fragments starting at the N-terminus could not be purified, we decided to purify fragments beginning at the putative globular domains of Trio, beginning with TrioN (residue 1284). We initially purified and imaged the Trio_1284-2638_ fragment, which included TrioN through SH3C (Fig. 1*C*). Micrographs revealed a globular particle with a diameter of ∼10 nm, but this particle did not exhibit any discernible features in 2D averages (Fig. S1). Larger Trio fragments including the Ig and kinase domains did not express. Therefore, we decided to proceed with functional experiments on the central region of Trio containing the catalytic domains and their associated SH3 domains.

### Basal and Gα_q_-stimulated GEF activity of Trio variants

**Figure 2:**
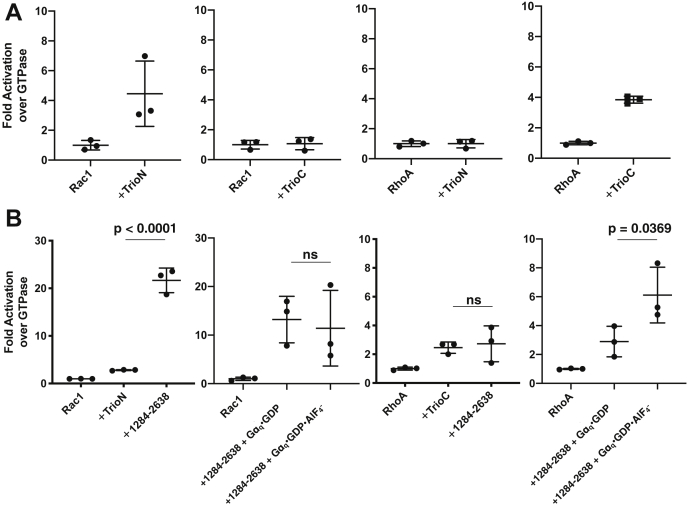
**GEF assays show enhanced Rac1 but not RhoA exchange in larger Trio fragments and that Gα**_**q**_**regulation of TrioC is insensitive to adjacent regions.***A*, control experiments showing that each Trio GEF module is selective for its given GTPase. *B*, basal activities of Trio_1284-2638_ compared to the isolated GEF modules and Gα_q_·GDP·AlF_4_^-^ stimulated Trio_1284-2638_. In each panel, Rac1 and RhoA exchange activity is shown in the *left* and *right* two plots, respectively. Note that in these assays Gα_q_·GDP·AlF_4_^-^ activates nucleotide exchange on RhoA but not Rac1. Scatter plots of GEF assays were normalized to rate of GTPase alone, N = 3 experiments in at least duplicate. Error bars indicate standard deviation. *p*-values are from a one-way ANOVA with Dunnett’s correction for multiple comparisons. GEF, guanine nucleotide exchange factor; ns, not significant.

**Figure 3:**
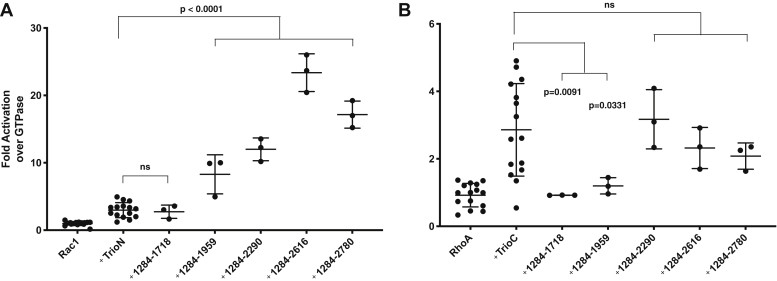
**GEF activity data for progressive C-terminal truncations of the middle region of Trio**. Panels (*A*) and (*B*) depict Rac1 and RhoA exchange experiments, respectively. *Box* and whisker plots of GEF assays were normalized to rate of GTPase alone (N = 3 experiments in at least duplicate). Error bars indicate standard deviation. *p*-values are from a one-way ANOVA with Dunnett’s correction for multiple comparisons. ns, not significant; GEF, guanine nucleotide exchange factor.

Using a Förster resonance energy transfer (FRET)-based GEF assay (Fig. S2), we first confirmed that the individual TrioN and TrioC GEF modules activate only their cognate substrates (Rac1 and RhoA, respectively, in our experiments) 3- to 4-fold (Fig. 2*A*). To test whether the presence of other regions affected this basal GEF activity, we compared the activity of the isolated GEF modules to that of Trio_1284-2638_. Trio_1284-2638_ had similar GEF activity on RhoA as TrioC in isolation, but 8-fold higher activity on Rac1 than TrioN in isolation (Fig. 2*B*). Gα_q_·GDP·AlF_4_^-^ enhanced Trio_1284-2638_ RhoA GEF activity to the same extent as TrioC (∼2.5-fold) but had no effect on Rac1 exchange activity (Figs. 2*B* and S3). Therefore, Gα_q_ seems agnostic to sequences outside the TrioC module. To identify the residues responsible for enhanced Rac1 GEF activity of TrioN, we generated a series of truncations (Fig. 3). These proteins started with residue 1284 at the N terminus of TrioN and ended at residue 1718 (after SH3N), 1959 (after a ∼240 residue linker region), 2290 (after TrioC), 2616 (after SH3C), and 2780 (after the Ig domain). Constructs larger than this did not express or behave well. Each variant was tested for activity *versus* TrioN or TrioC in paired experiments (Fig. 3). Constructs ending at residue 1959 or later displayed 3- to 7-fold enhanced GEF activity on Rac1 relative to TrioN alone, with Trio_1284-2616_ having the highest rate (Fig. 3*A*), similar to the rate observed for Trio_1284-2638_. All the variants that included the TrioC GEF module had similar activity on RhoA relative to TrioC alone (Fig. 3*B*).

### Cryo-EM analysis of the Trio_1284-1959_–Rac1 complex

**Figure 4:**
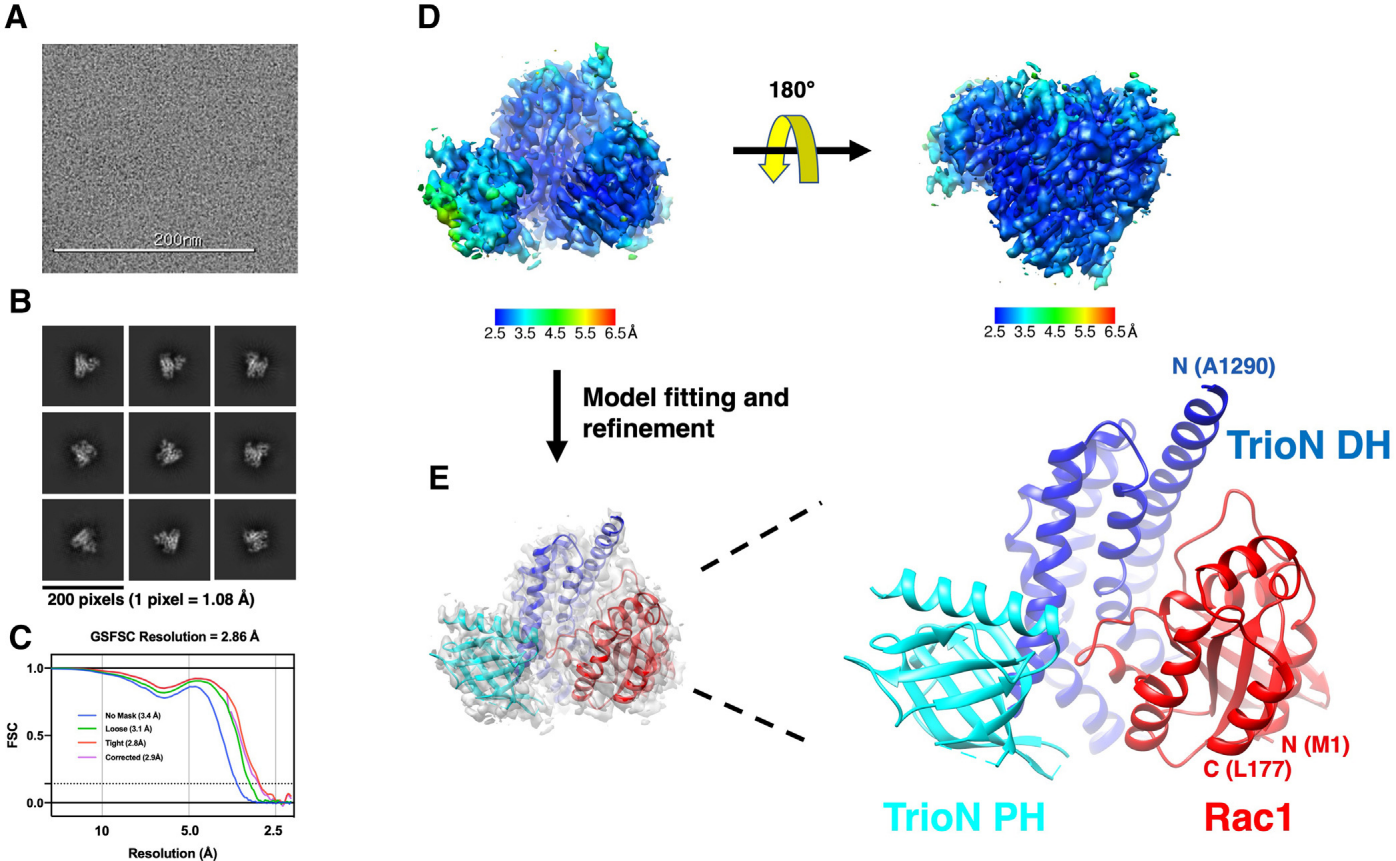
**Cryo-EM reconstruction of the Trio**_**1284-1959**_**–Rac1 complex.***A*, representative cryo-EM micrograph. *B*, representative 2D classes. *C*, resolution estimation of the Trio_1284-1959_—Rac1 cryo-EM reconstruction based on the FSC=0.143 criterion with different masking options. *D*, cryo-EM map of Trio_1284-1959_-Rac1. Local resolution of the cryo-EM map was estimated in cryoSPARC. *E*, model fitting and refinement. A starting model generated from the crystal structure of the TrioN–Rac1 complex (PDB entry 2NZ8) was fit into the cryo-EM density map and then refined using Coot and PHENIX. The DH and PH domains of TrioN are shown in *blue* and *cyan*, respectively. Rac1 is shown in *red*. DH, Dbl homology; EM, electron microscopy; PH, pleckstrin homology.

To gain structural insight into the mechanism of GEF enhancement, we decided to use a single particle cryo-EM approach. We chose Trio_1284-1959_ for this analysis because its molecular weight (78 kDa) was potentially large enough to get a high resolution reconstruction and also because it was the most well-behaved and highest yielding Trio variant. Furthermore, it is composed of a relatively simple system of three structural domains (DH, PH, and SH3N), which would facilitate modeling if the resolution of the reconstruction were low. The Trio_1284-1959_–Rac1 complex was first profiled by negative stain EM (Fig. S4), yielding particles about 10 nm in diameter that seemed to have better detail than particles of Trio_1284-2638_ alone (Fig. S1). Cryo-EM single particle analysis ultimately yielded a 2.9 Å map (Fig. 4), which is unusually high resolution for what ended up being a ∼55 kDa structure (not considering regions that ended up disordered). To fit the map, the crystal structure of the TrioN–Rac1 complex (PDB entry 2NZ8) was placed into the map, then subjected to multiple rounds of model building and real space refinement (Fig. 4, Table 1, and Fig. S5). The final model contains residues 1 to 177 of Rac1, and residues 1284 to 1594 of Trio. However, there was no obvious density for any domain or residues C terminal to the Trio PH domain. Comparison of the complex, which may be the first of a RhoGEF DH/PH module by single particle cryo-EM, with that of the TrioN–Rac1 crystal structure reveals few differences (Fig. S5). More residues of the PH domain are ordered in the cryo-EM structure, and the domain is rotated about 4° with respect to the DH domain relative to that of the crystal structure. The register of the β5 strand of the PH domain is different, but the new cryo-EM structure agrees with the register of the model in the crystal structure of TrioN alone (PDB entry 1NTY). Density for the N-terminal helix of the cryo-EM DH domain extends outward by eight additional residues, and residues 26 to 31 in Switch 1 of Rac1 adopt a markedly different conformation. Most of the differences between the models seem to be a consequence of lattice contacts in the 2NZ8 structure, which occur near Switch 1 of Rac1 and at the hinge region of the DH/PH module. Furthermore, if the N-terminus of Trio in 2NZ8 were extended as a helix as far as it is in the cryo-EM structure, it would cause a steric clash with a lattice contact. This suggests that a cryo-EM structure, even at a lower nominal resolution of 2.9 Å, can potentially yield more detail and, perhaps, a more native conformation than a 2 Å crystal structure.

**Table 1:** Cryo-EM data collection, processing, and model fitting statistics

Trio_1284-1959_–Rac1 complex EMDB-25153 PDB 7SJ4	
Data collection and processing	
Magnification	81,000
Voltage (kV)	300
Electron exposure (e–/Å^2^)	55
Defocus range (μm)	−0.5∼−2.2
Pixel size (Å)	0.54
Symmetry imposed	C1
Initial particle images (no.)	2,066,058
Final particle images (no.)	922,202
Map resolution (Å)	2.86
FSC threshold	0.143
Refinement	
Initial model used (PDB code)	2NZ8
Model resolution (Å)	2.0
Map sharpening *B* factor (Å^2^)	−147
Model composition	
Nonhydrogen atoms	3909
Protein residues	481
Water	24
*B* factors (Å^2^)	
Protein	74.7
Ligand	46.1
R.m.s. deviations	
Bond lengths (Å)	0.002
Bond angles (°)	0.432
Validation	
MolProbity score	1.14
Clashscore	3.47
Poor rotamers (%)	0.93
Ramachandran plot	
Favored (%)	98.73
Allowed (%)	1.27
Disallowed (%)	0

### Analysis of the Trio_1284-1959_–Rac1 complex by HDX-MS

**Figure 5:**
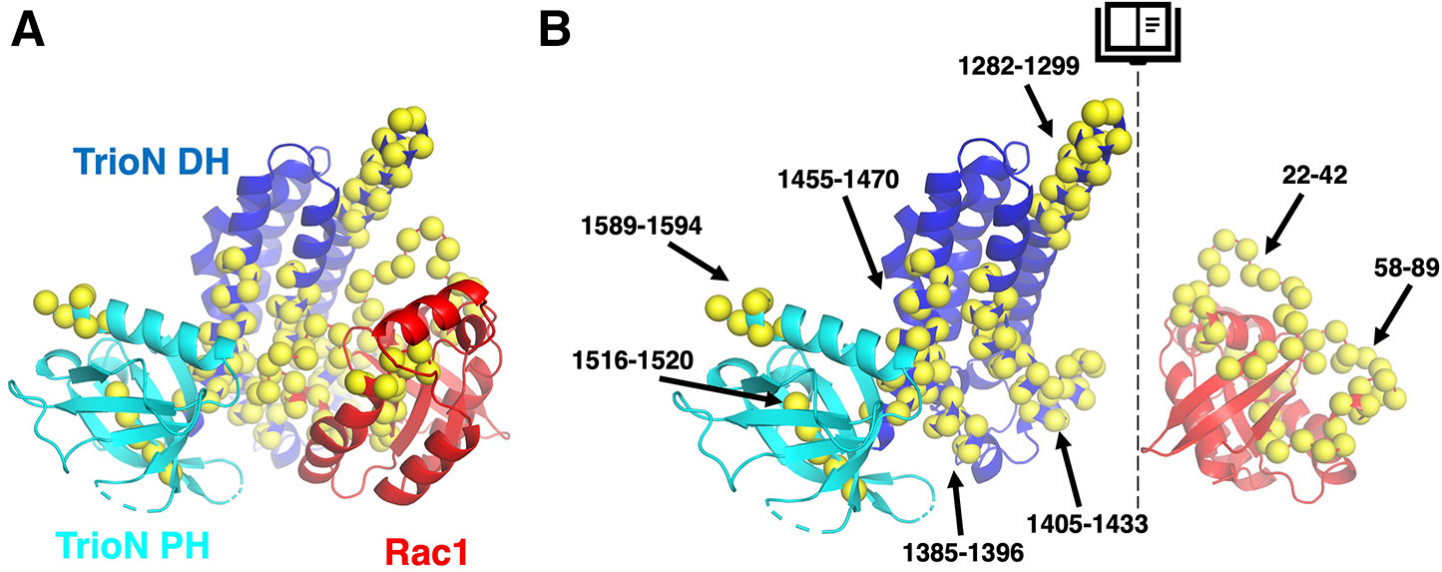
**Mapping the interaction interface between Trio**_**1284-1959**_**and Rac1.***A*, location of decreased HDX signal in the complex structure of Trio_1284-1959_-Rac1 modeled using the crystal structure of the TrioN-Rac1 complex (PDB 2NZ8). Cα positions of residues with decreased HDX signal are indicated with a *yellow sphere*. The DH and PH domains of TrioN are shown in *blue* and *cyan*, respectively. Rac1 is *red*. *B*, “open book” view of TrioN and Rac1 showing regions with decreased HDX signal, wherein both subunits in panel A have been rotated 90° in opposite directions around the *vertical axis* (*dashed lines*). DH, Dbl homology; HDX, hydrogen-deuterium exchange; GEF, guanine nucleotide exchange factor; PH, pleckstrin homology.

**Figure 6:**
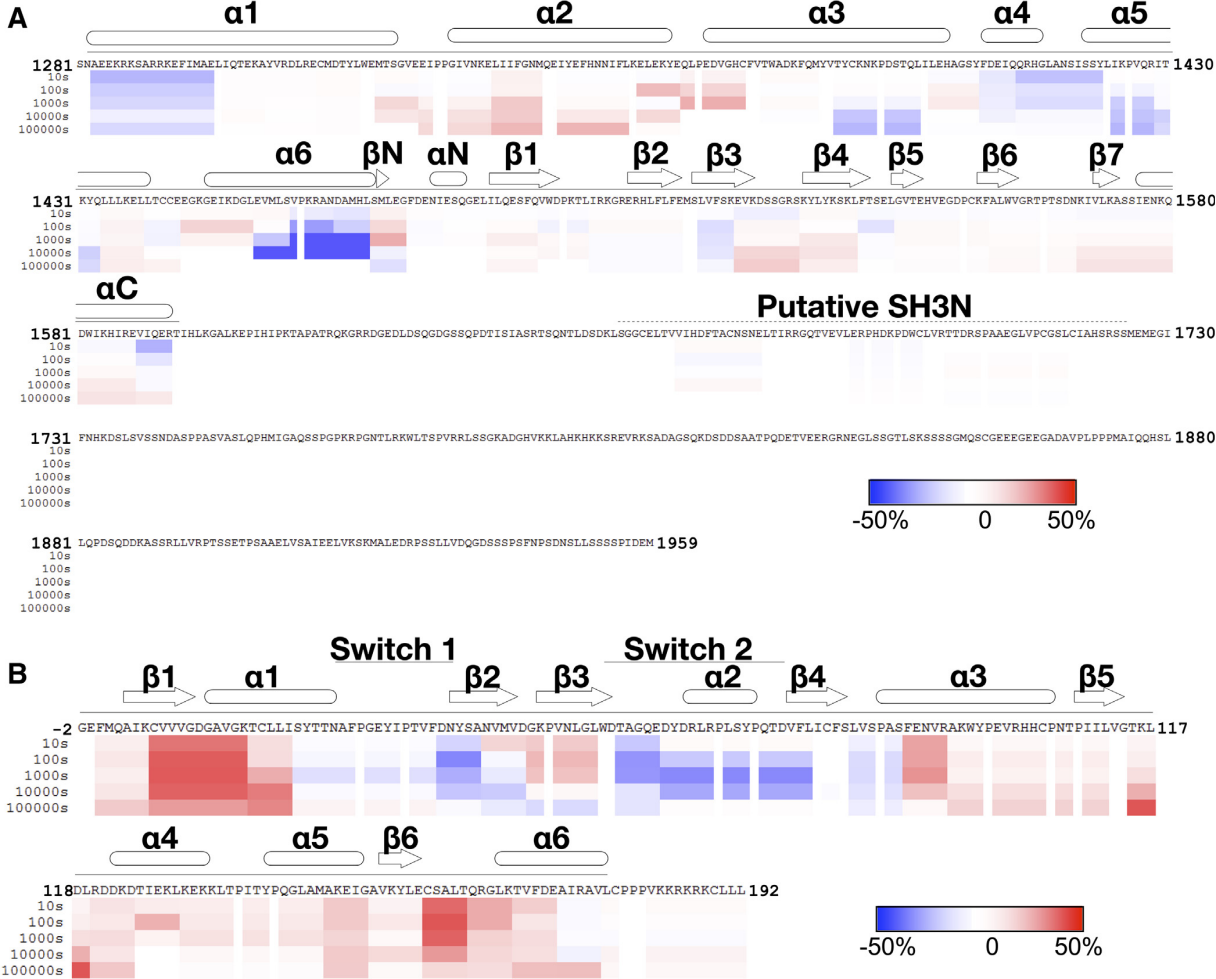
**The HDX-MS profile of Trio**_**1284-1959**_**suggests that residues within the 1718 to 1959 region do not form a stable interaction with either Rac1 or TrioN.***A*, HDX-MS difference map of Trio_1284-1959_ alone *versus* in complex with Rac1. *B*, HDX-MS difference maps of Rac1 alone *versus* in complex with Trio_1284-1959_. The extent of deuterium incorporation is shown as colored *rectangles*, indicating less exchange (*blue*) to more exchange (*red*) of the complex relative to the isolated subunits. Time points are shown on the *left* in seconds. Protein primary sequences are shown with secondary structure above each profile, with α helices shown as *rounded cylinders*, β strands as *arrows*, and a *straight line* indicating ordered regions of the Trio_1284-1959_-Rac1 cryo-EM structure. EM, electron microscopy; HDX-MS, hydrogen-deuterium exchange mass spectroscopy.

We next collected HDX-MS data on Trio_1284-1959_, Rac1·GDP, and their complex to see if there were regions in the C terminus of the Trio fragment beyond the PH domain whose dynamics changed upon complex formation, as one might expect if they interacted with either regions in the fragment or Rac1. The HDX-MS map of Trio_1284-1959_ alone (Fig. S6) showed the anticipated exchange pattern for peptides derived from the DH and PH domains of the GEF module (residues 1284–1594), with lower exchanging regions corresponding to those observed in crystal structures and surface residues and loops displaying higher exchange. Following the GEF module, the only peptides showing any solvent protection are those roughly corresponding to the SH3N domain. The linker region we showed to confer activation (residues 1719–1959) are likely disordered because they all exchange within 10 s after the start of the experiment. The corresponding map for Rac1·GDP (Fig. S8*A*) was consistent with crystal structures of the molecule. In HDX-MS difference maps comparing Trio_1284-1959_ and Rac1·GDP alone to the Trio_1284-1959_–Rac1 complex (Figs. 5, 6 and S6–S8), the overall HDX profile is as one might expect from the structure of the TrioN–Rac1 complex. Residues from each protein that form the interface exhibited a decrease in exchange in the complex consistent with their sequestration from solvent, including Trio_1284-1959_ residues in α1, α3, α4, α5, and most strongly α6. Residues in switch 1 and switch 2 of Rac1 which form the binding surface and interact with the DH domain in Trio_1284-1959_ also displayed lowered exchange rates in the complex, indicative of sequestration from solvent. On the other hand, Rac1 residues in β1, α1, β5, α4, and the β6-α6 loop which contact the nucleotide when bound exhibited an increase in exchange, consistent with an expected increase in dynamics associated with the nucleotide-free state of Rac1 when bound to the DH domain. Trio residues 1719-1959 showed no change in deuterium incorporation upon complex formation (Fig. 6), suggesting that residues in this region only transiently interact with Rac1 or the TrioN GEF module.

### Analysis of Trio_1284-1959_ enhanced activity using site-directed mutagenesis

**Figure 7:**
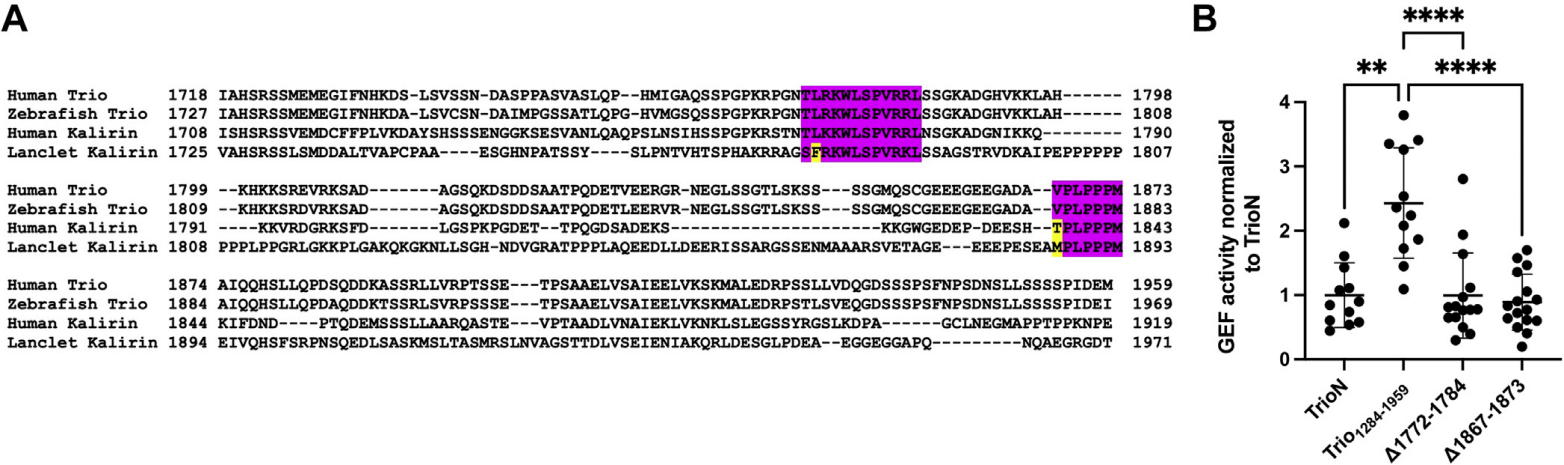
**Identification of linker regions that contribute to GEF activity on TrioN.***A*, representative sequences from a multisequence alignment of Trio homologs and Kalirin orthologs. There are two conspicuous spans of residues that are highly conserved across the 13 vertebrate and invertebrate sequences analyzed (*purple* and *yellow* highlights corresponding to identical/highly conserved and conserved positions, respectively). Uniprot entries for the shown sequences are O75962 (human Trio Isoform1), F1Q551 (zebrafish Trio Isoform 1), O60229 (human Kalirin Isoform 1), and A0A6P4YLV6 (amphioxus Kalirin). *B*, deletion of either conserved span decreased exchange on Rac1 to a level similar to TrioN alone. Each dot represents an individual experiment from N = 12 total experiments. Error bars indicate standard deviation. ∗∗*p* = 0.0045; ∗∗∗∗*p* < 0.0001.

Because we could not detect any region within residues 1595 to 1959 that might contribute to enhanced Rac1 GEF activity by TrioN by cryo-EM or HDX-MS, we assessed if there were spans of residues in this region that were conserved across metazoan life and found two prominent ones: a basic and hydrophobic motif and a proline-rich motif at Trio residues 1772 to 1784 and 1867 to 1873, respectively (Fig. 7*A*). We created and purified deletion variants of each span (Trio_1284-1959_,_Δ1772-1784_ and Trio_1284-1959_,_Δ1867-1873_) and compared their GEF activity against those of TrioN and Trio_1284-1959_ (Fig. 7*B*). Deletion of either conserved span led to reduction of Rac1 GEF activity to the same level as that of TrioN, indicating that both elements are essential for the increased catalytic proficiency of Trio_1284__–__1959_.

## Discussion

**Figure 8:**
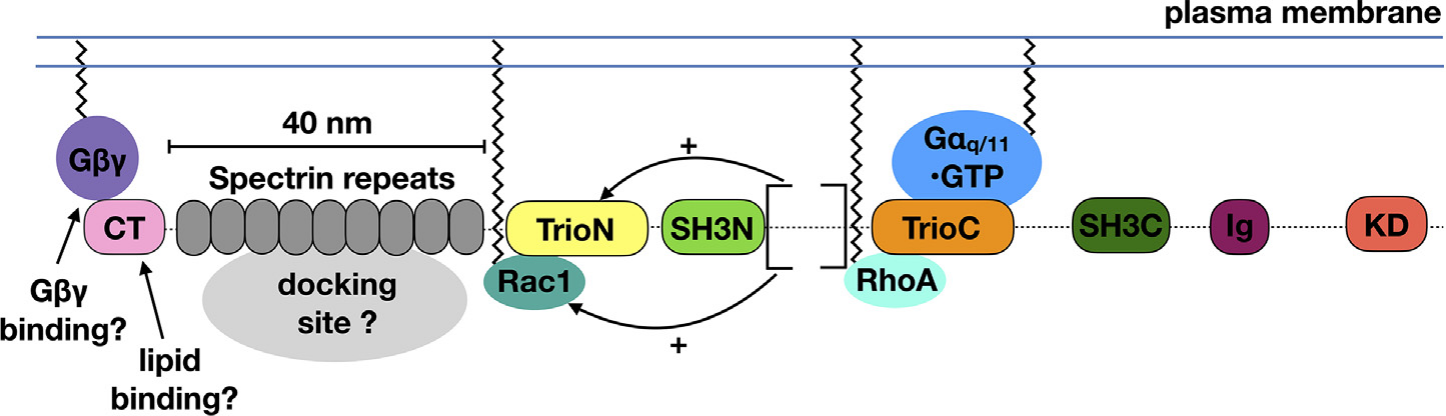
**Hypotheses for Trio regulation.** From left to right, the CT domain may interact with Gβγ or specific lipids on the plasma membrane and cooperate with the spectrin region to spatially regulate Rac1 signaling. The spectrin repeat region may also serve as a docking site for other signaling proteins or complexes. The 1718 to 1959 region following the SH3N domain (bracketed) facilitates nucleotide exchange on Rac1 *via* a transient interaction with either TrioN, Rac1, or SH3N. Gα_q/11_ binds to the TrioC module to directly enhance RhoA nucleotide exchange and likely helps to localize Trio to the plasma membrane, which in turn enhances Rac1 exchange as observed in cells. *Serrated lines* represent lipid modifications, and *dashed lines* represent putative unstructured regions (not drawn to scale). CT, Cral/Trio.

We expected that learning more about Trio domains accessory to the GEF modules would provide details about autoregulation of GEF activity, thereby creating opportunities for discovery of therapeutics that selectively target these unique regulatory mechanisms. To this end, we characterized large Trio fragments using negative stain and cryo-EM, HDX-MS, and FRET-based nucleotide exchange assays. The N-terminal region of Trio (represented by Trio_MBP-61-1594_), which is dominated by nine spectrin repeats, forms a particle with an extended conformation as visualized by negative stain EM (Fig. 1*B*). Thus, we speculate, as have others, that the spectrin repeats may serve to maintain distance between the domains present at each end, in this case a lipid or protein binding activity of the CT domain and the Rac1/RhoA exchange activity of TrioN (Fig. 8). Distance between a lipid binding site and GEF activity may be important in the neurite outgrowth process wherein the extension of neurites requires a particular distance between plasma membrane components and the actin cytoskeleton (28, 33). Indeed, Trio’s involvement in neurite outgrowth requires the spectrin repeat region, TrioN GEF activity, and the SH3N domain (28). The extended spectrin repeat region may also provide a docking interface for other proteins such as supervillin (34). Higher resolution insights into this region by EM would however require somehow reducing conformational heterogeneity.

Conversely, the central region of Trio (residues 1284–2638) was globular in negative stain EM micrographs, but no high-resolution features were present in the resulting 2D averages, suggesting that this portion of Trio may consist of self-associating regions that are loosely organized, at least without other interaction partners. Consistent with this idea, we found that larger Trio constructs exhibited faster Rac1 exchange rates than the TrioN GEF module alone. Activated Gα_q_ in the form of Gα_q_∙GDP∙AlF_4_^-^ did not affect the Rac1 nucleotide exchange of any fragment tested (Figs. 2 and S3) but did activate fragments containing TrioC ∼2.5-fold (Figs. 2 and S3). Thus, Gα_q_ binding does not seem to allosterically affect TrioN GEF activity and allosterically activates the TrioC module regardless of its context. Because Gα_q_ binding was observed to stimulate both Rac1 and RhoA exchange *via* Trio in cells (6, 7), it seems likely that Gα_q_ stimulates Rac1 turnover through Trio simply by driving the association of Trio to the plasma membrane where Rac1 and RhoA are localized (Fig. 7).

The minimum fragment required in our study to see significant Rac1 turnover rate enhancement spanned residues 1284 to 1959, including TrioN, SH3N, and a 240-residue low complexity linker that extends to the beginning of the TrioC module (Fig. 3). Because Trio_1284-1718_ did not display enhanced exchange rate, we concluded that the 240-residue region between SH3N and TrioC (residues 1719–1959) was critical for rate enhancement. However, analysis of the Trio_1284-1959_–Rac1 complex using cryo-EM did not find evidence for any ordered portion of the 1719 to 1959 region that might facilitate complex formation (Figure 4, Figure 5, Figure 6). Furthermore, the 1719 to 1959 region exhibited high dynamics by HDX-MS regardless of Rac1 binding (Figs. 6*A* and S6 and S7). Thus, if elements within the 1719 to 1959 region directly facilitate GEF activity, it seems they only do so transiently. Sequence analysis of Trio and its close ortholog Kalirin across vertebrates and invertebrates reveal two highly conserved spans in the 1719 to 1959 region (Fig. 7*A*). Deletion of either span eliminated enhancement of GEF activity by Trio_1284-1959_, indicating that they may be playing evolutionarily important roles, but the underlying mechanisms remain unknown. One span (1772–1784) is both basic and hydrophobic and may transiently interact with a complementary region on TrioN or Rac1. The other conserved span (1867–1873) is proline rich and contains a PXXP motif, suggesting that it could bind to one (or both) SH3 domains in Trio. A peptide corresponding to the analogous span was tested for binding to SH3N in Kalirin, but its affinity was much weaker than other PXXP containing peptides tested from the N-terminal half of the enzyme. Furthermore, the interaction of these peptides with SH3N was reported to be inhibitory toward GEF activity (26). We did not detect an analogous decrease in GEF activity when comparing TrioN to longer fragments also containing the SH3N domain. Instead, we documented profoundly higher activity. Whether the SH3N domain is required for this boost in GEF activity in Trio is not yet known. Our opposing results from those reported for Kalirin could be due to distinct experimental conditions and/or different protein fragments being tested (or even a fundamental difference between Kalirin and Trio). Regardless, both sets of data suggest that there exist complex intramolecular interactions mediated by various motifs within the low complexity linker regions in these proteins which likely contribute to spatial and temporal regulation of Trio in cells. These interactions present an opportunity to modulate Trio function for therapeutic benefit.

## Experimental procedures

### Cloning

Human Trio cDNA consisting of a DNA sequence corresponding to residues 61 to 3097 in pcDNA3.1 was described previously (7). All Trio protein constructs are described with numbering relative to isoform 1 in UniProt entry O75962 and were designed using insight from the XtalPred Server (35) and Clustal Omega (36). DNA regions for larger Trio constructs were amplified using the KOD polymerase kit (EMD Millipore) following manufacturer’s instructions, which was necessary for productive high-fidelity amplification. Individual Trio domains were amplified using Q5 polymerase (NEB). Deletions were generated using overlap extension PCR. Inserts were generated using PCR reactions with ligation-independent cloning handles on 5′ and 3′ ends, purified using a PCR cleanup kit (Qiagen), and inserted into the pMCSG9 vector using the ligation-independent cloning protocol (37).This allowed for *E. coli* expression and affinity purification of MBP-Trio fusion variants (N-6xHis-MBP-TEV-Trio-C). Constructs were confirmed using Sanger sequencing of plasmid DNA purified using a Mini-prep kit (Qiagen). The RhoA and Rac1 constructs were described previously (38) and consist of residues 1 to 193 of human RhoA and 1 to 192 of human Rac1. The Gα_q_ construct used was also described previously (39).

### Protein expression and purification

RhoA and Rac1 were purified as described (38). Plasmids encoding Trio variants were transformed into Rosetta (DE3) pLysS *E. coli* cells (Novagen) and grown in terrific broth (EMD Millipore Sigma) with 100 μg/ml ampicillin or carbenicillin plus 50 μg/ml chloramphenicol at 37 °C with 200 rpm shaking. Once an *A*_600_ of 0.6 to 0.8 was reached, expression of N-terminally tagged fusion proteins was induced using 0.5 mM isopropyl β-D-1-thiogalactopyranoside, and cells were transferred to 20 °C with 200 rpm shaking for 20 to 24 h. Cells were then harvested at 5000*g* for 15 min, and cell pellets were flash-frozen if not immediately prepared. Cell pellets were vortexed and resuspended using a dounce homogenizer in ice-cold lysis buffer consisting of 20 mM 4-(2-hydroxyethyl)-1-piperazineethanesulfonic acid (HEPES) pH 8.0, 200 mM NaCl, 2 mM dithiothreitol (DTT), 5% (v/v) glycerol, 0.001 mM leupeptin, 1 mM lima bean trypsin inhibitor, 0.1 mM phenylmethylsulfonyl fluoride, and 5 mM EDTA. Resuspended cell solution was then lysed using a handheld VirSonic 100 Sonicator (Boston Laboratory Equipment) for five 30-s pulses at 18 W on ice. Lysate was then centrifuged at 40,000 rpm in a Beckman Optima L-90K ultracentrifuge (Beckman-Coulter) to remove insoluble material. The soluble fraction was then filtered through a 0.45 μm filter and loaded onto Roche cOmplete His-tag resin (Roche 5893682001) equilibrated with lysis buffer. Two aliquots of 10 column volumes of lysis buffer containing 10 mM imidazole were used to wash the column. The recombinant protein was then eluted using lysis buffer plus 200 mM imidazole. The elution fractions containing the desired protein were then incubated with 5% (w/w) tobacco etch virus protease to cleave the N-terminal expression tag, and the mixture was dialyzed against a buffer containing 20 mM HEPES pH 8.0, 200 mM NaCl, and 2 mM DTT. After removal of the MBP fragment by passage through a cOmplete resin column, Trio constructs were dialyzed against 20 mM HEPES pH 8.0, 10 mM NaCl, and 2 mM DTT overnight at 4 °C. Proteins were subjected to anion exchange chromatography using a 5 ml HiTrap Q HP column (GE 17115401) using a buffer of 20 mM HEPES pH 8.0 and 2 mM DTT with a gradient of 10 mM to 1000 mM NaCl over 100 ml. Desired fractions were concentrated in a 30 or 50 kDa cutoff concentrator and loaded on either a 24 ml Superose 6 or a Superdex 200 column (GE) equilibrated with 20 mM HEPES pH 8.0, 200 mM NaCl, and 2 mM DTT. Desired fractions from SEC were concentrated to 1 to 2 mg/ml and flash frozen in liquid N_2_. For formation of Trio–GTPase complexes, Trio variants were mixed with >2-fold molar excess of GTPase in a buffer containing 0.1 mM EDTA to drive complex formation. Complexes were incubated at 4 °C for >30 min and then loaded on an S200 column equilibrated with 20 mM HEPES pH 8.0, 200 mM NaCl, 2 mM DTT, and 0.1 mM EDTA. Fractions containing complex were concentrated in a 30 kDa cutoff concentrator, and protein concentration was determined using A_280_.

### Negative stain EM data collection and processing

Negative staining of Trio samples was performed similarly to that previously described (40). Purified protein samples were applied to a glow discharged formvar coated copper grid (Electron Microscopy Sciences FCF400-Cu-50) for 1 min. Grids were first blotted against filter paper, then processed by two rounds of dipping into ddH_2_O followed by blotting, and then two rounds of dipping into 0.75% (w/v) uranyl formate stain followed by blotting. The grids were then dipped into another drop of stain for 1 min. Finally, grids were blotted dry and dried using a vacuum line. The resulting grids were evaluated for stain quality, contrast, particle quality, and particle spread using a Morgagni 100 kV transmission electron microscope (TEM). Micrographs were collected on it using a CCD camera at 2.1 Å/pixel at a nominal magnification of 22,000× using an exposure time of 1 s. Optimal grids were taken for further imaging on the Tecnai T12 120 kV TEM operated using the Leginon automated data collection system (41) and a nominal magnification of 67,000×, 1 s exposure time, and a CCD camera at 1.68 Å/pixel. Particles were picked for 2D class averaging using the *cis*TEM software suite (42). For the Trio_1284-2638_ construct, 4000 particles were averaged into 20 classes. For the Trio_1284-1959_–Rac1 complex, 15,000 particles were averaged into 50 classes. Use of fewer classes for the Trio_1284-1959_–Rac1 complex did not improve detail.

### FRET-based guanine exchange factor assay

FRET was used to assess the nucleotide exchange activity of Trio variants (13, 43). In a 384-well black low-volume round-bottom microplate (Corning 4514), 2 μM RhoA·GDP or Rac1·GDP was incubated with 50 nM GEF for 5 min at room temperature in freshly prepared nucleotide exchange buffer containing 20 mM HEPES pH 8.0, 200 mM NaCl, 2 mM DTT, and 10 mM MgCl_2_. Immediately before measurement, 1 μM 2´/3′-O-(N-methyl-anthraniloyl)-guanosine-5′-triphosphate (MANT-GTP) (Jena Biosciences) was added to a final assay volume of 20 μl. The mixture was then excited at 280 nm, and fluorescence emission at 450 nm was read in 5 s intervals on a SpectraMax M5 plate reader (Molecular Devices) for 5 to 10 min. Resulting curves were fit to a linear regression model to derive the observed kinetic constant k_obs_ and then compared to that of matched rates of GTPase alone and GTPase + control GEF (TrioN or TrioC). For Gα_q_ activation assays, Gα_q_·GDP was added at 200 nM, and the assay was run in the presence or absence of 30 μM AlCl_3_ and 10 mM NaF, which generates the active Gα_q_·GDP·AlF_4_^-^ complex. For GEF assays shown in Figure 7*B*, the protocol is same as mentioned above except the fluorescence curves were measured on a Flexstation 3 plate reader (Molecular Devices) and fitted to a one-phase exponential association model (Y=Y_0_ + SPAN∗(1-exp(-κ∗X)) with SPAN constrained to be shared for all datasets in a single experiment.

### Statistical analysis

FRET nucleotide exchange assays were performed in at least three independent experiments. For assays in Figures 2 and S3, the three experiments were performed using aliquots from the same protein preparation. For assays in Figure 3, the first experiment was performed using one protein preparation, the second and third were performed using protein from a second purification. For assays comparing basal GEF activities in Figures 2 and 3, k_obs_ values were normalized to rate of GTPase alone to generate fold activation values for each variant. For Gα_q_·GDP·AlF_4_^−^ activation assays in Figure 2*B*, k_obs_ values were normalized to rate of GTPase alone to generate fold activation values for each. For GEF assays shown in Figure 7*B*, FRET experiments were performed with protein from single preparations of TrioN and Trio_1284-1959_ as negative and positive controls, respectively, but two different preparations of each deletion mutant. These assays were normalized to the rate of Rac1 + TrioN, and Fig. S3 were normalized to the rate of GTPase + GEF + Gα_q_·GDP (no AlF_4_^−^ added). Statistical significance was assessed using a one-way ANOVA test with a post-hoc Dunnett’s test for multiple comparisons. In basal GEF assays, each Trio variant is statistically compared to either TrioN or TrioC. Normalized k_obs_ in the presence of Gα_q_·GDP·AlF_4_^−^ for each variant was statistically compared to k_obs_ in the presence of Gα_q_·GDP. Errors are presented as standard deviations from the mean. Analyses were performed using GraphPad Prism, version 7.0.

### Hydrogen-deuterium exchange mass spectrometry

HDX-MS experiments were carried out as previously described (13). The optimal quench solution to give best sequence coverage map of the Trio_1284-1959_·Rac1 complex contained 6.4 M guanidinium hydrochloride (Gu-HCl), 1 M Tris(2-carboxyethyl)phosphine, and 0.1 M glycine, pH 2.4. All exchange stock solutions were prepared on ice and contained 2.5 mg/ml of Trio_1284-1959_, 2.5 mg/ml of Trio_1284-1959_·Rac1, and 2.2 mg/ml of Rac1 in 8.3 mM Tris pH 7.2 and 150 mM NaCl. The exchange experiments were initiated by adding 2 μl of stock solutions to 4 μl of D_2_O buffer containing 8.3 mM Tris, 150 mM NaCl, pD 7.2. The exchange solutions were kept at 0 °C for 10, 100, 1,000, 10,000, and 100,000 s and then quenched by the addition of 9 μl of the quench solution and incubated on ice for 10 min. The quenched samples were further diluted with 45 μl of 0.1 M glycine, pH 2.4, 16.6% (v/v) glycerol to drop the Gu-HCl concentration below 1 M and stored on dry ice. All samples were passed through a pepsin column for enzymatic digestion, and the resulting peptides were collected on a trap column (Optimize Tech, OptiTrap, 0.2 × 2 mm). Liquid chromatography separations were performed on an Agilent C18 column (Poroshell 120, 0.3 × 35 mm, 2.7 μm) with a linear acetonitrile gradient and analyzed using an OrbiTrap Elite Mass Spectrometer (Thermo Fisher Scientific), which was tuned up for HDX experiments (44). Data acquisition and peptide identification were done by Xcalibur 3.0 and Proteome Discoverer 1.3 (Thermo Fisher Scientific). Deuterium uptake was calculated with HDXaminer (Sierra Analytics, LLC), by correcting the back-exchange with control samples. Ribbon map coloring were generated using MATLab by combining deuterium uptake information of overlapping peptides.

### Cryo-EM data collection and processing

The Trio_1284-1959_·Rac1 complex was prepared at 2.6 μM concentration and n-dodecyl-β-D-maltoside was added to a final concentration of 0.08 mM. Samples were frozen using the Vitrobot automated grid freezing system (FEI) on UltraAuFoil R (1.2/1.3) 300-mesh gold grids (Electron Microscopy Sciences). The Vitrobot was set to 4 °C, 100% humidity, 3.5 s blot, and a force of 10. Cryo-EM samples were screened using the 200 kV Talos F200C Glacios TEM, and grids with evenly distributed particles were clipped using C-clips (Electron Microscopy Sciences) for the following cryo-EM data collection using the 300 kV Titan Krios G1 TEM with a post-GIF K3 direct electron detector at 0.54 Å/pixel with a nominal magnification of 81,000×. Each of the 3514 movies collected has 40 consecutive frames, which were used to generate a motion-corrected micrograph by MotionCor2 (45). The contrast transfer function (CTF) of each micrograph was estimated using the Patch-CTF estimation package in cryoSPARC with default settings. After rejection of micrographs with poor CTF fitting, 2817 micrographs were used for particle picking and extraction using cryoSPARC (46). Successive rounds of reference-free 2D classification were performed to select protein particles while eliminating junk particles, yielding 1,216,361 particles. After application of the Rebalance 2D step, 904,361 particles were selected for producing two *ab initio* models, which were used to classify the 1,216,361 particles in the 3D heterogenous refinement. Finally, 922,202 particles were used for homogenous refinement to obtain a 3D map. Two reconstructed half maps were used for resolution estimation using the Fourier shell correlation criterion of 0.143, and a sharpened map was used for model building in RELION (47). The crystal structure of the N-terminal DH/PH cassette of TrioN complexed with Rac1^16^ (PDB: 2NZ8) was first rigid-body fit into the cryo-EM map using UCSF Chimera (48) and subjected to manual model building and real-space refinement using Coot (49) and PHENIX (50). Model validation was performed by MolProbity (51). Coordinates and maps were deposited in the PDB as entry 7SJ4 and the EMDB as entry 25,153.

## Data availability

Plasmids and other reagents are available upon reasonable request to J. J. G. T. The model for the Trio_1284-1959_–Rac1 complex has been deposited as PDB entry 7SJ4 and as EMDB entry EMD-25153.

## Supporting information

This article contains supporting information.

## Conflict of interest

The authors declare that they have no conflicts of interest with the contents of this article.

## Supporting information

Supporting information

TrioN-Rac1-coot_037

cryosparc_P47_J439_005_volume_map_sharp(2)
