# Supplementary material for: Structural/functional studies of Trio provide insights into its configuration and show that conserved linker elements enhance its activity for Rac1
Source: J Biol Chem. 2022 Jun 30;298(8):102209. doi: 10.1016/j.jbc.2022.102209 (PMC9372627; doi:10.1016/j.jbc.2022.102209)
Supplement: Supporting information [file mmc1.docx]

**SUPPORTING INFORMATION FOR**

**Structural/functional studies of Trio provide insights into its configuration and show that conserved linker elements enhance its activity for Rac1**

**Authors:** Sumit J. Bandekar^1,2^, Chun-Liang Chen^4^, Sandeep K. Ravala^4^, Jennifer N. Cash^3^, Larisa V. Avramova^4^, Mariya V. Zhalnina^4^, J. Silvio Gutkind^5^, Sheng Li^6^, and John J. G. Tesmer^4, *^

**Affiliations:**

^1^Department of Medicinal Chemistry, University of Michigan, Ann Arbor, MI, 48109, USA.

^2^Life Sciences Institute, University of Michigan, Ann Arbor, MI, 48109, USA.

^3^Department of Molecular and Cellular Biology, University of California-Davis, Davis, CA, 95616, USA.

^4^Departments of Biological Sciences and of Medicinal Chemistry and Molecular Pharmacology, Purdue University, West Lafayette, IN, 47907, USA.

^5^Department of Pharmacology and Moores Cancer Center, University of California, San Diego, San Diego, CA 92093, USA

^6^Department of Medicine, University of California San Diego, La Jolla, CA 92093, USA.

*To whom correspondence should be addressed: jtesmer@purdue.edu

**Supplemental Figure S1: Negative stain EM analysis of the Trio_1248-2638_ fragment.** Top, raw micrograph. Middle, domain diagram of Trio_1248-2638_. Bottom, representative 2D class averages segregating 4,000 particles into 20 classes. Data was collected using Leginon on the Tecnai T12 microscope at a nominal magnification of 67,000x, and processed using *cis*TEM.

**
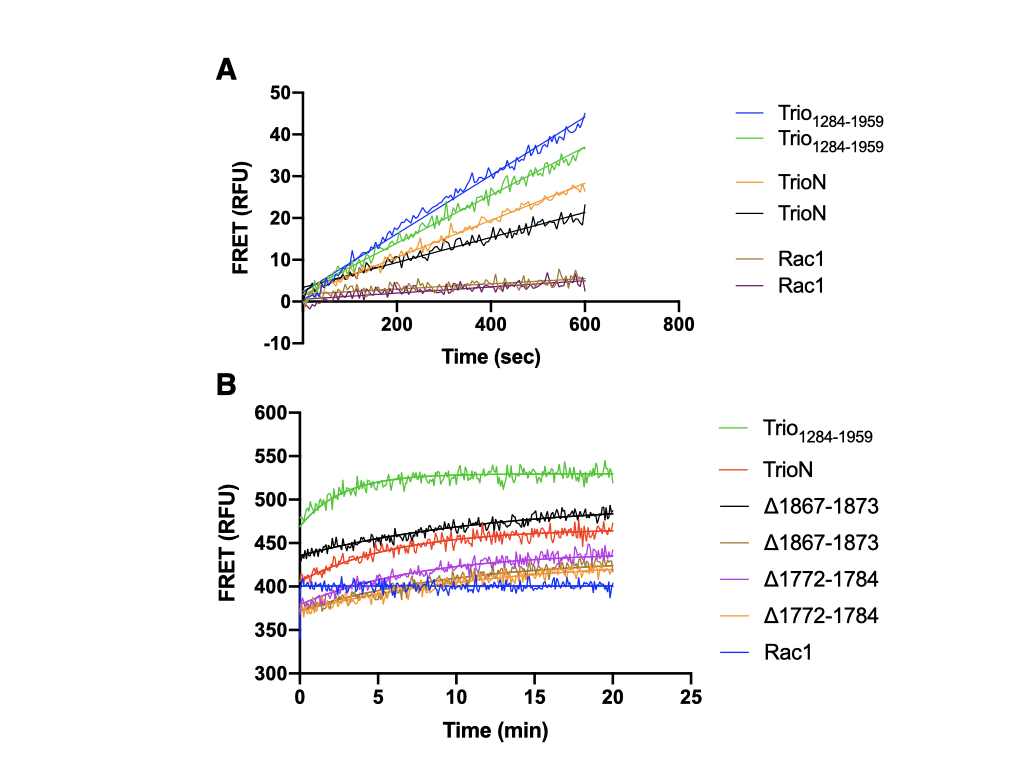
**

**Supplemental Figure S2:** **Representative raw nucleotide exchange data and fits used to derive k_obs_ and relative rates_._** (A) Examples of raw data and linear fits used to generate main text Figures 2 and 3, and Supplementary Figure S3. Samples were run in duplicate. (B) Example of raw data and exponential fitting used to generate Figure 7B. Here the variants being tested were run in duplicate, but control reactions (Rac1, TrioN, and Trio_1284-1959_) were run in singlicate. Different fits were used in these examples due to the use of slightly different conditions and different instrumentation, which yielded different curves.


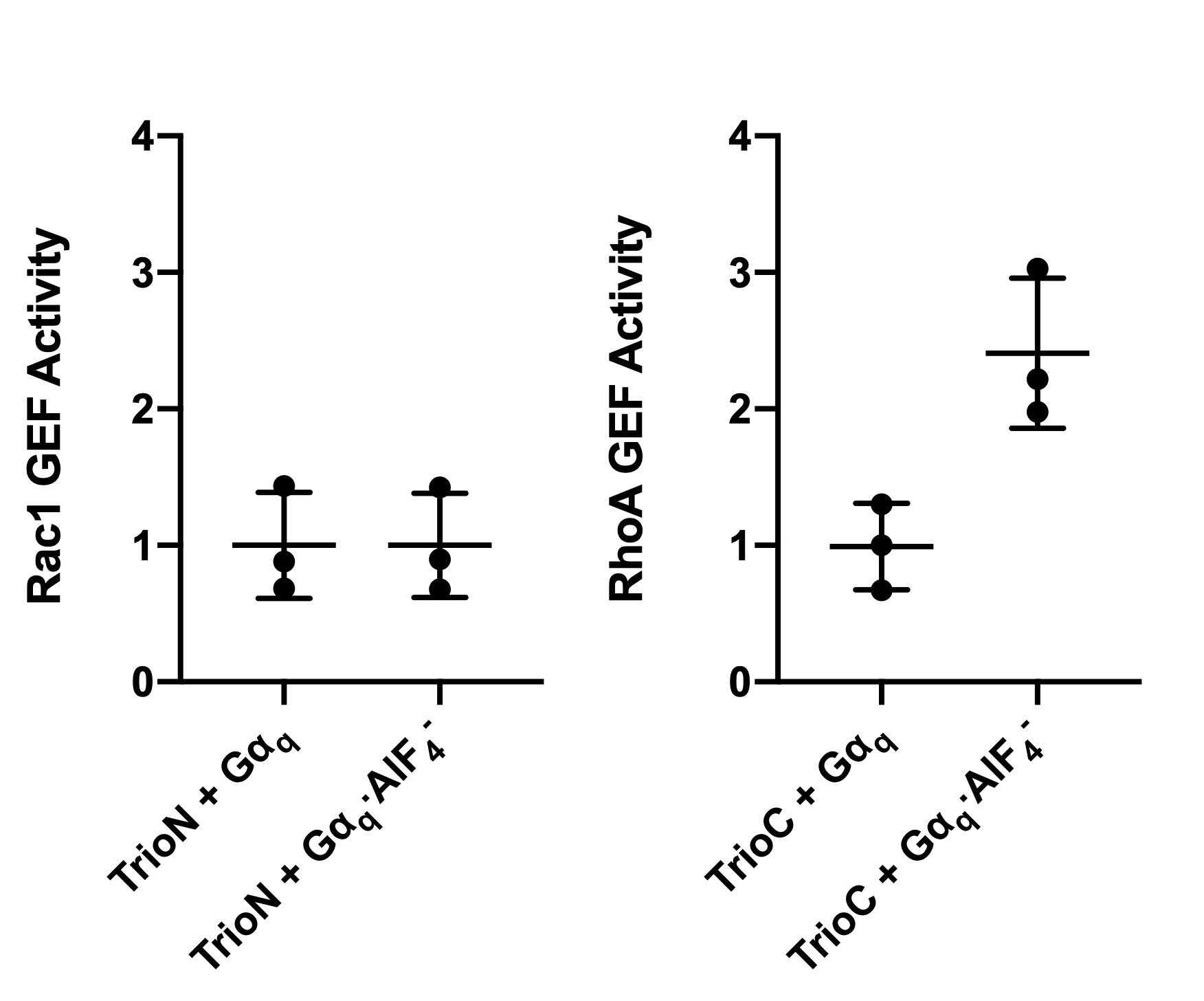


**Supplemental Figure S3: Gα_q_ only activates variants containing the TrioC module.** Left and right plots depict GEF activity on Rac1 or RhoA, in the presence or absence of either deactivated (GDP) or activated (GDP·AlF_4_^-^) Gα_q_, respectively. GEF assays in each individual experiment were normalized to rate of condition with no AlF_4_^-^. N=3 experiments. Error bars indicate standard deviation.

**Supplemental Figure S4:** **Negative stain EM analysis of the Trio_1284-1959_–Rac1 complex**. This complex forms a particle of about 10 nm in diameter. Left, representative raw micrograph of particles. Right, the result of 2D classification segregating 15,000 particles into 50 classes. Data was collected using Leginon on a Tecnai T12 microscope at a nominal magnification of 67,000x, and processed using cisTEM.

**
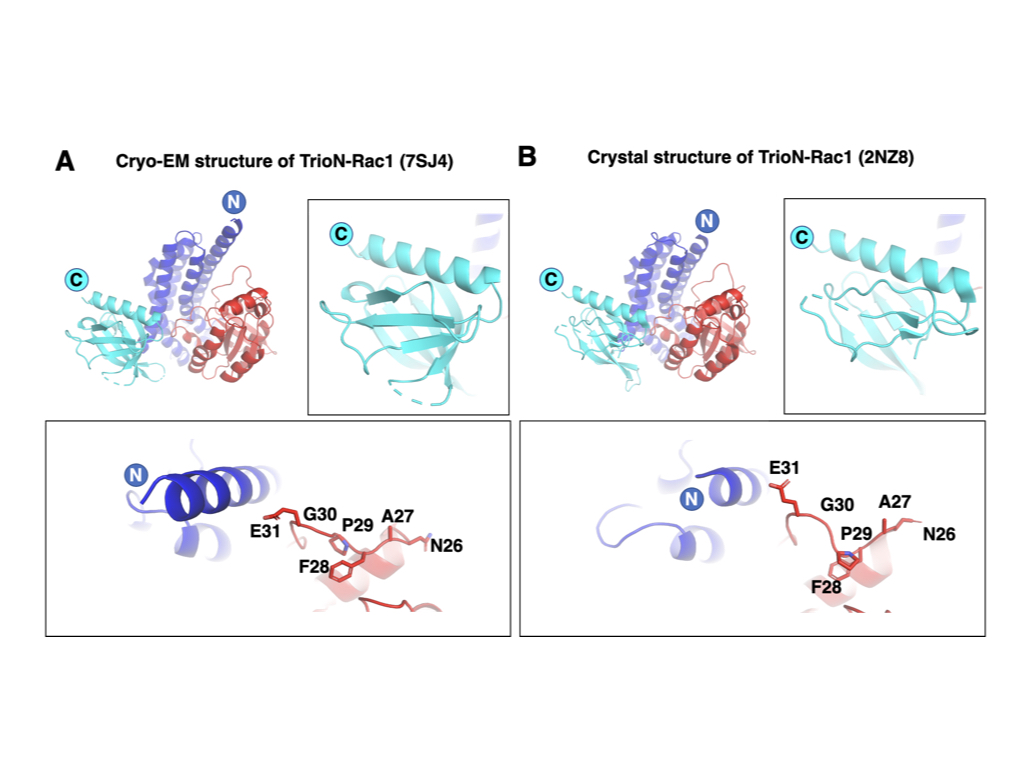
**

**Supplemental Figure S5: Comparison of the Trio_1284-1959_-Rac1 cryo-EM structure with TrioN-Rac1 crystal structure.** (A) Cryo-EM structure of the TrioN-Rac1 complex (upper-left). The DH and PH domains of TrioN are colored in blue and cyan, and Rac1 in red. The N- and C-termini of TrioN are indicated. Upper-right: a close-up view of the PH domain. Bottom: a close-up view of the region around residues 26-31 in Switch 1. Residue side chains are shown as sticks. (B) Analogous views of the crystal structure of the TrioN-Rac1 complex (PDB entry 2NZ8). The crystal structure of TrioN-Rac1 aligned to the cryo-EM structure with an RMSD value of 0.68 Å for 238 Cα atoms.


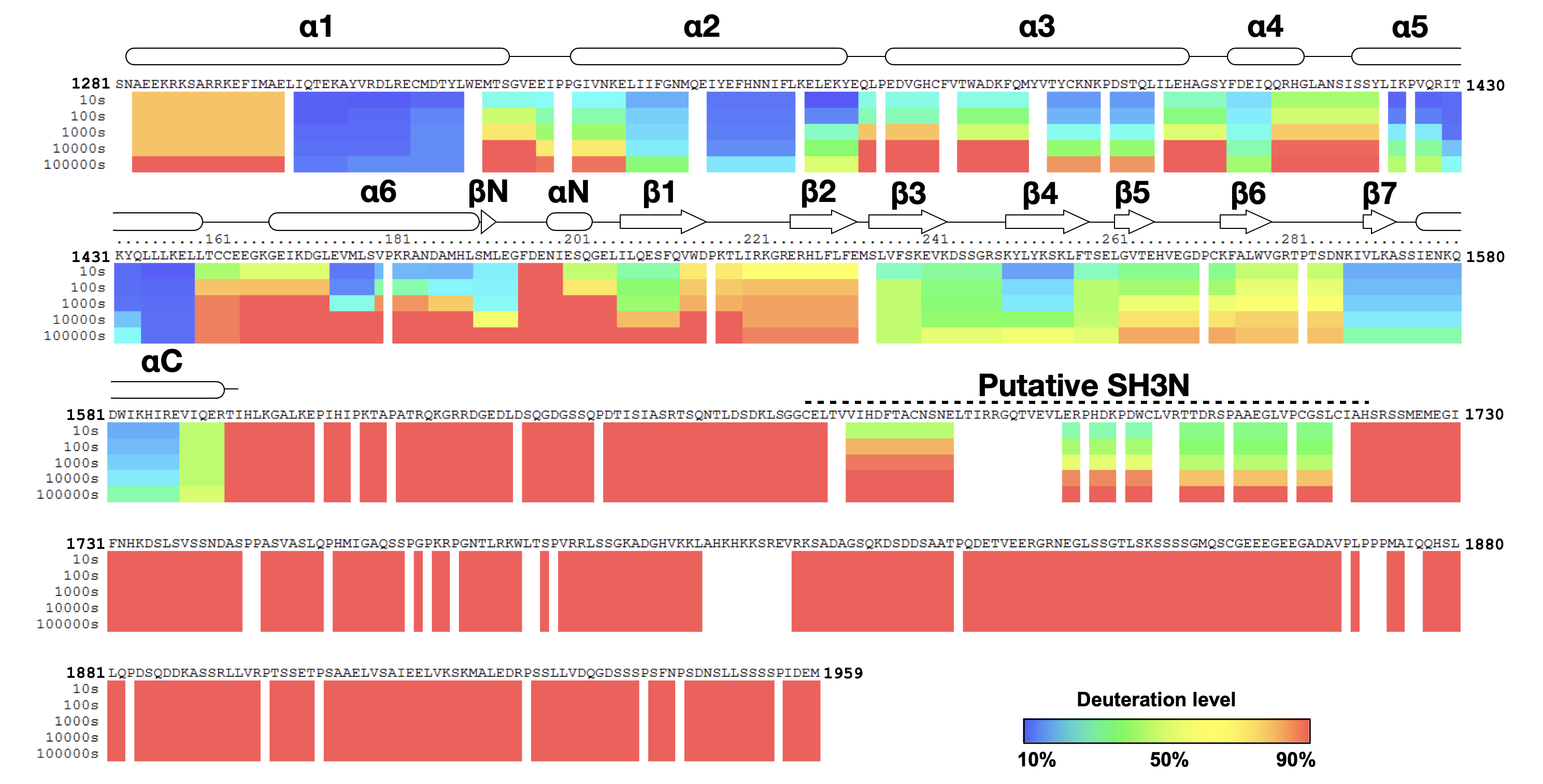


**Supplemental Figure S6: HDX-MS profile of Trio_1284-1959_ alone.** The extent of deuterium incorporation is shown as colored rectangles, ranging from less exchange (blue) to more exchange (red). Time points are shown on the left. Protein primary sequence is shown with secondary structure highlighted above the profile, with α helices shown as rounded cylinders, β strands as arrows, and a straight line indicating ordered regions of the Trio_1284-1959_-Rac1 cryo-EM structure.

**
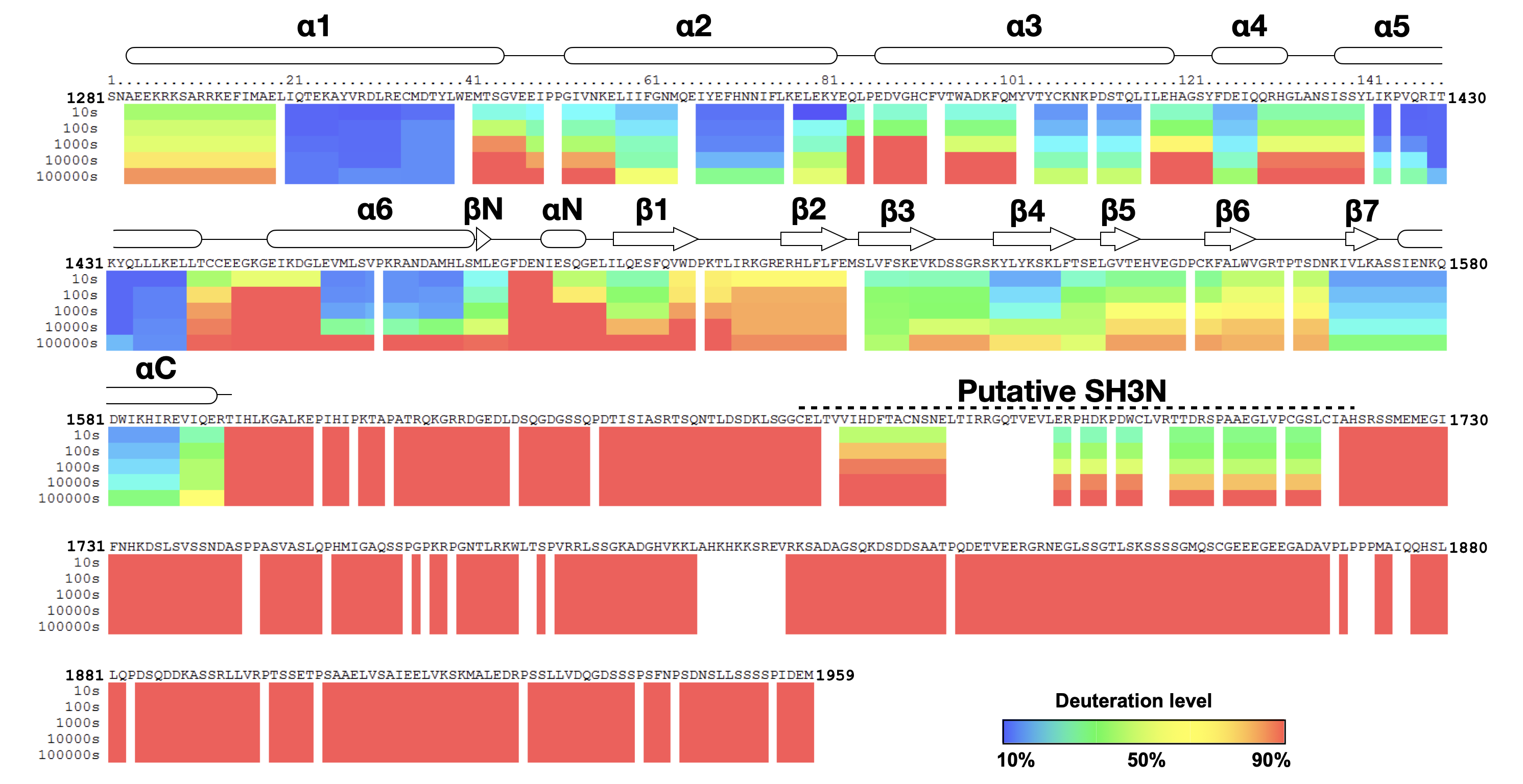
**

**Supplemental Figure S7: HDX-MS profile of Trio_1284-1959_ in complex with Rac1.** The extent of deuterium incorporation is shown below the primary sequence as colored rectangles, ranging from less exchange (blue) to more exchange (red). Time points are shown on the left. Protein primary sequence is shown with secondary structure highlighted above, with α helices shown as rounded cylinders, β strands as arrows, and a straight line indicating ordered regions of the Trio_1284-1959_-Rac1 cryo-EM structure.

**
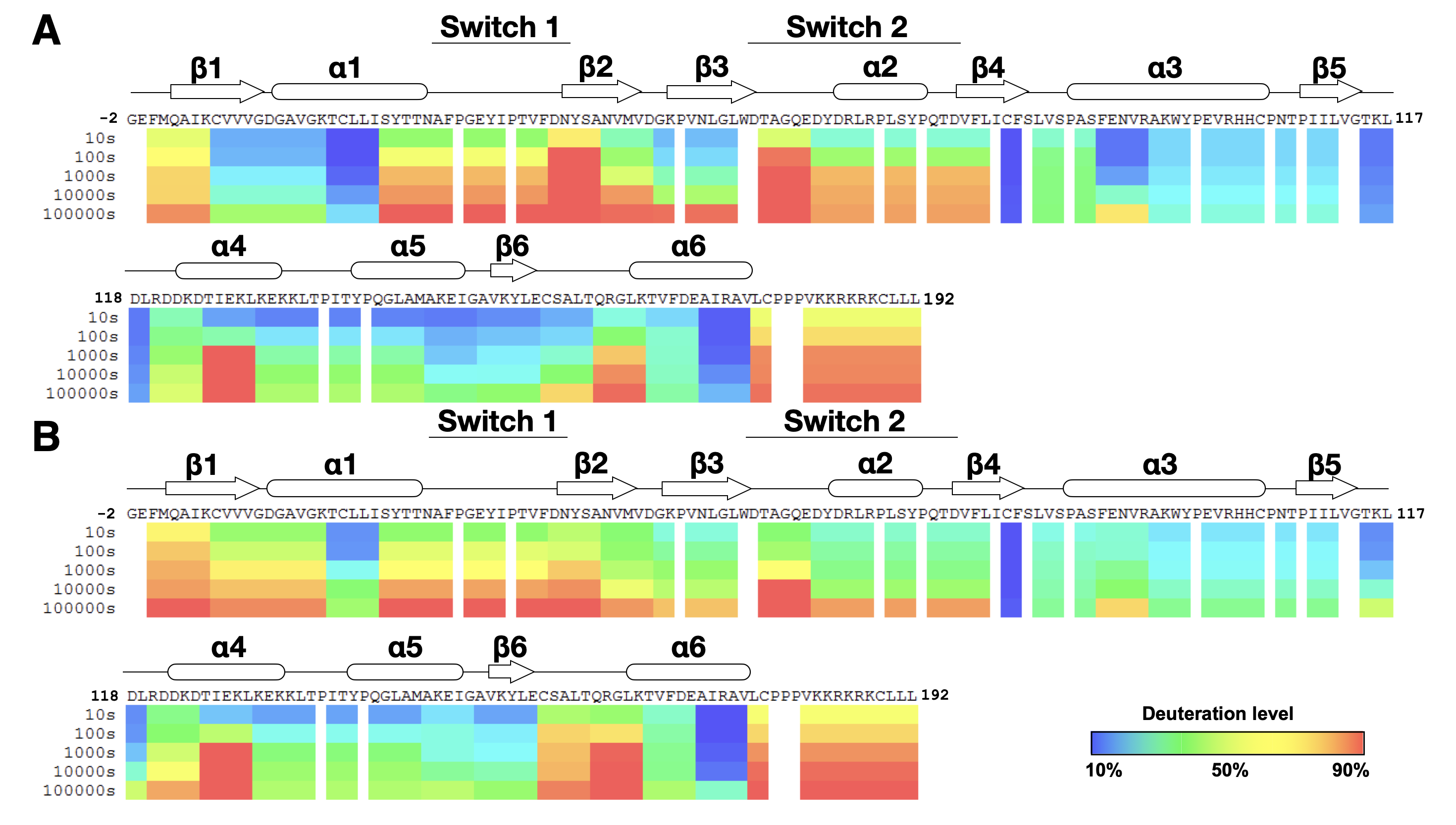
**

**Supplemental Figure S8: HDX-MS profile of (A) Rac1 alone and (B) in complex with Trio_1248-1959_.** The extent of deuterium incorporation is shown below the primary sequence as colored rectangles, ranging from less exchange (blue) to more exchange (red). Time points are shown on the left. Protein primary sequences are shown with secondary structures highlighted above, with α helices shown as rounded cylinders, β strands as arrows, and a straight line indicating ordered regions of the Trio_1284-1959_-Rac1 cryo-EM structure.
